# Synthesis of Copper Nanostructures for Non-Enzymatic Glucose Sensors via Direct-Current Magnetron Sputtering

**DOI:** 10.3390/nano12234144

**Published:** 2022-11-23

**Authors:** Sabrina State (Rosoiu), Laura-Bianca Enache, Pavel Potorac, Mariana Prodana, Marius Enachescu

**Affiliations:** 1 Center for Surface Science and Nanotechnology, Politehnica University of Bucharest, Splaiul Independentei 313, 060042 Bucharest, Romania; 2 Department of General Chemistry, Faculty of Applied Chemistry and Materials Science, Politehnica University of Bucharest, Splaiul Independentei 313, 060042 Bucharest, Romania; 3 Academy of Romanian Scientists, Splaiul Independentei 54, 050094 Bucharest, Romania

**Keywords:** direct-current magnetron sputtering, Cu nanocolumns, non-enzymatic, glucose sensing

## Abstract

In this paper, Cu nanocolumnar structure electrodes are synthetized using a clean and easy-to-scale-up direct-current magnetron sputtering (DC-MS) technique for non-enzymatic glucose sensing. The nanocolumnar structure increases the active surface area of the deposit, with the nanocolumns showing a mean size diameter of 121.0 nm ± 27.2 and a length of 2.52 µm ± 0.23. A scanning transmission electron (STEM) analysis shows the presence of Cu and a small amount of Cu_2_O. The behavior of the electrodes in alkaline environments and the electrochemical affinity of the Cu nanocolumns (CuNCs) towards the electro-oxidation of glucose are investigated using cyclic voltammetry (CV). After performing CV in NaOH solution, the columnar structures present corrosion products containing Cu_2_O, as revealed by STEM and X-ray diffraction (XRD) analyses. The amperometric responses of the CuNCs to the successive addition of glucose show a linear range up to 2 mM and a limit of detection of 5.2 µM. Furthermore, the electrodes are free from chloride poisoning, and they are insensitive to dopamine, uric acid, ascorbic acid, and acetaminophen at their physiological concentrations.

## 1. Introduction

Glucose determination is crucial in various fields, such as clinical medicine, wastewater treatment, and the food industry. In 2019, over 1.5 million deaths were directly cause by diabetes, as established by the World Health Organization [1]. The accurate monitoring of glucose in blood is essential in the diagnosis and treatment of diabetes. Therefore, over the past few decades, a significant effort has been made to develop reliable, fast, and economic glucose sensors [2]. The first glucose biosensor was reported by Clark and Lyons in 1962, based on the glucose oxidase (Gox) enzyme [3]. However, the use of enzymatic sensors is problematic since the activity of Gox is susceptible to pH values and humidity, it is sensitive to temperature variation and toxic chemicals, in addition to the high cost of the enzyme and the difficulty of immobilizing it [4]. To overcome these disadvantages, non-enzymatic biosensors have been explored. 

Metallic nano- and micro-structures have been used in enzyme-free glucose detection. Copper has attracted considerable attention due to its electrocatalytic activity towards glucose oxidation, low cost, and high electrical conductivity. In addition, it does not suffer from chloride poisoning, abundant in human blood, as compared to noble metals [5]. One-dimensional copper nanostructures are of great interest since they offer efficient electron transfer along the 1D direction and good mechanical strength [5]. Various non-enzymatic glucose sensors based on copper 1D nanostructures have been reported. Zhang [6] and Mihai [7] prepared copper nanowires (CuNWs) using a soft template made out of polycarbonate. Even though the morphology of the nanowires was controlled by the template, the main drawback was related to the template removal. Other authors synthetized CuNWs by using wet chemical methods [8]. Zhang et al. prepared copper nanowires (CuNWs) using a reduction method with CuNO_3_ as a precursor, toxic hydrazine as a strong reducing agent, and ethylenimine as a capping agent [5]. Wonjoo Na and co-authors also synthetized CuNWs using a reduction method, utilizing glucose and hexadecylamine [9]. However, the glucose-assisted route requires long reaction times. The authors reported 12 h for the stirring of copper chloride, glucose, and hexadecylamine, in addition to another 24 h for autoclaving at 120 °C. Furthermore, Cu nanobelts [10], CuO nanofibers [11], CuO nanoroads [12], CuO nanowires [2,13], and other 1D copper composite nanostructures (such as CuNW–carbon nanotubes [14] and CuNW-reduced graphene oxide [15,16,17]) have been reported as potential glucose sensors. Generally, wet chemistry is used in their synthesis, most often involving hazardous chemicals and elevated temperatures, which are not suitable for large-scale preparation. Plus, after the nanomaterial is synthetized, commonly, it is further immobilized on the electrode surface, introducing an extra step in the glucose sensor fabrication process. Therefore, it is still challenging to develop a cost-effective, reproducible, and environmentally friendly method that produces the copper nanostructure directly on the electrode surface for glucose sensing. 

To overcome the above-mentioned drawbacks, magnetron sputtering (MS) is a robust method used to prepare copper nanocolumns directly on the surface of electrodes without byproducts, and it is easy to scale up [18,19]. Direct-current magnetron sputtering (DC-MS) is a one-step physical fabrication process consisting of a target material that is bombarded with ionized gas molecules causing the atoms from the target to be sputtered onto the substrate [20]. Several parameters, such as surface roughness, surface temperature, and adatom surface mobility and geometry, influence the film grown [21]. The morphology of the sputter-deposited film is described using the structural zone model proposed by Thornton depending on the substrate temperature and the pressure of the inert gas [22]. The columnar grain structure of thin films has been observed for several metals obtained via sputtering, Cu being among them [23]. Craig and Harding reported the effects of the argon pressure and glass substrate temperature of sputtered copper film [24]. A well-defined columnar morphology of the film was observed via sputtering at a high pressure (9.7·10^−1^ mbar) and a low substrate temperature (T/T_m_ = 0.23). This columnar morphology helps to increase the surface area of the coating, making it suitable for glucose detection. Recently, Siampour et al. investigated the use of Cu nanocolumns, obtained via glancing angle deposition (GLAD) using a thermal evaporation system on FTO substrate, as non-enzymatic glucose sensors [25]. However, in general, the films obtained via thermal evaporation exhibit poor uniformity, a high level of impurities, and limited scalability. 

Herein, we report, for the first time, the use of copper nanocolumns (CuNCs) obtained using DC-MS for non-enzymatic glucose determination. The CuNCs were prepared at a fixed argon pressure of 4.0 × 10^−2^ mbar and room temperature on glassy carbon substrate (GC). The deposits were characterized using scanning electron microscopy (SEM) and scanning transmission electron microscopy (STEM). Their activity towards glucose detection was investigated in 50 mM NaOH solution using cyclic voltammetry (CV) and chronoamperometry. Additionally, the anti-interference properties of the sensor were determined for different compounds usually found in human blood.

## 2. Materials and Methods

### 2.1. Reagents

Sodium hydroxide (Sigma-Aldrich, Steinheim, Germany), potassium hexacyanoferrate (II) trihydrate (≥99%, Sigma-Aldrich, Steinheim, Germany), potassium hexacyanoferrate (III) (≥99%, Sigma-Aldrich, Steinheim, Germany) and anhydrous D-(+)-Glucose (99%, Alfa Aesar, Kandel, Germany) were used as received. For interference tests, sodium chloride (lach:ner, Késmárk, Budapest), dopamine (99%), uric acid (99%), ascorbic acid (99.7%), and acetaminophen (98%) were purchased from Acros Organics (Geel, Belgium), and sucrose (≥99.5%) and fructose (≥99%) were from Sigma-Aldrich (Steinheim, Germany). All solutions were prepared using deionized water with a conductivity of 0.5 µS/cm.

### 2.2. Preparation of the Non-Enzymatic Glucose Electrodes

Prior to the deposition of the metals, glassy carbon electrodes (5 mm in diameter, Metrohm) were polished with a 1 µm diamond paste (Buehler) and a 0.3 µm alumina suspension solution, followed by deionized water and ethanol. On the same electrodes, copper was sputtered using a direct-current magnetron sputtering machine (Mantis Deposition Ltd., Thame, UK). The copper target has a purity of 99.99% and was used as a received from Kurt J. Lesker company (Dresden, Germany). The substrate was not heated; however, it was rotated at 7 rpm, and the distance between the copper target and the substrate was fixed at 23 cm. The chamber was firstly evacuated to a pressure of 4 × 10^−5^ mbar and then filled with a constant flux of argon (Linde 5.0, ≤2 O_2_ vpm) until a pressure of 4 × 10^−2^ mbar was established. The sputtering was performed at a power of ~90 W, and the average deposition rate was 0.06 nm/s. The thickness of the deposit was monitored using a quartz balance. The copper was DC-sputtered for three hours.

In order to improve the adherence of the DC-sputtered copper to the glassy carbon substrate in alkaline environments, a thin layer of gold was deposited on it. Gold was evaporated using the e-beam evaporation technique on an M-EV mini e-beam Evaporator (Mantis Deposition Ltd., Thame, UK) system. The deposition was performed under vacuum at a pressure of 4 × 10^−5^ mbar and a power of 44 W with an average deposition rate of 0.02 nm/s until 30 nm was reached. The same quartz balance was used to monitor the thickness of the film.

### 2.3. Characterization of the Non-Enzymatic Glucose Electrodes

The surface morphology of the electrodes was characterized using a Hitachi SU 8230 (Hitachi High-Tech Corporation, Tokyo, Japan) scanning electron microscope (SEM), equipped with an energy-dispersive X-ray detector from Oxford Instruments (Oxford, UK). Furthermore, the SEM images were analyzed using “Image J” software (LOCI, University of Wisconsin) in order to determine the length and diameter distribution of the columns. Over 500 columns were measured, and the corresponding histograms were fit with a normal distribution. High-resolution scanning transmission electron (HR-STEM) micrographs were acquired for the same location at the same magnification using three different types of detectors, namely, a surface electron detector (SEM), a high-angle annular dark-field detector for atomic mass phase contrast (ZC-phase contrast), and a bright-field detector for transmission electron imaging (TEM), and all of them were obtained using a Hitachi HD-2700 system (Hitachi High-Tech Corporation, Tokyo, Japan) operating at 200 kV. The X-ray diffraction spectra were recorded in a Rigaku SmartLab (Tokyo, Japan) using Cu Kα radiation (λ = 0.15406 nm) at 45 kV in a 2θ range of 5°–90°.

### 2.4. Electrochemical Investigations

To investigate the applicability of the electrodes for glucose detection, cyclic voltammograms (CVs) and chronoamperometric measurements were performed on a three-electrode cell with a platinum plate as a counter electrode (Metrohm, Herisau, Switzerland), Ag/AgCl (Metrohm, Herisau, Switzerland) as a reference, and glassy carbon (or a copper strip for comparison) covered with the deposited materials as a working electrode (5 mm diameter, s = 0.196 cm^2^, Metrohm, Herisau, Switzerland). The cyclic voltammograms were recorded starting at the open-circuit potential (OCP) in the −1 to +1 V range. The chronoamperometry experiments were performed at a constant potential of +0.7 V in 5 mL of 50 mM NaOH solution. The electrode potential was stabilized over 500 s in the electrolyte prior to the successive addition of glucose. Furthermore, at least three replicates were performed for each experiment using the PARSTAT 4000 system (Ametek, Berwyn, PA, USA) controlled with VersaStudio software (2.1 version, Ametek, Berwyn, PA, USA).

## 3. Results and Discussion

### 3.1. Characterization of Cu Nanocolumns 

**Figure 1:**
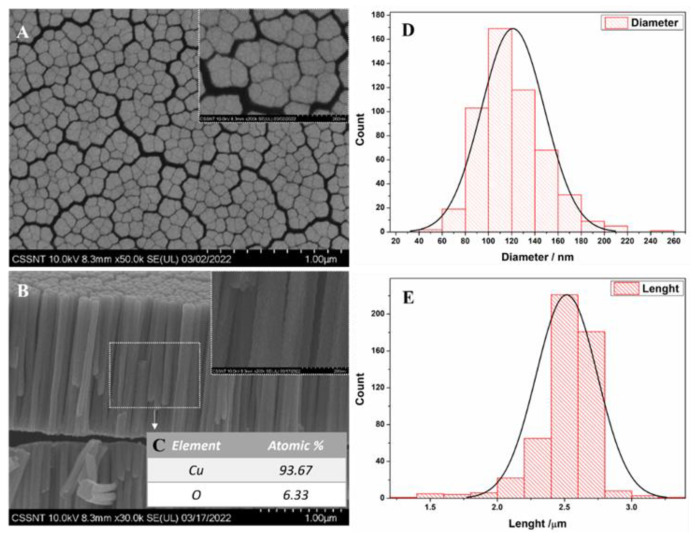
SEM images of CuNCs: (**A**) top-view; (**B**) cross-section; (**C**) elemental analysis; (**D**,**E**) their corresponding diameter and length distribution.

The SEM images displayed in Figure 1 present the copper obtained via DC magnetron sputtering on a glassy carbon (GC) substrate under an argon atmosphere at a pressure of 4.0 × 10^−2^ mbar. It can be observed from the top-view SEM analysis that the resultant deposit presents a nodular morphology. On the cross-section, a well-defined columnar morphology is observed. According to the Thornton classification, this morphology can be assigned to Zone I of the theoretical model (T/T_m_ = 0.22 and 30 mTorr), which is characterized by open boundaries defining columnar grains [22,26]. A morphology similar to that shown here has also been reported in the literature for magnetron-sputtered copper deposits [24,26,27]. The substrate temperature and the argon pressure strongly affect the morphology of copper deposits. For a similar T/T_m_ value, Craig and Harding reported that increasing the argon pressure results in a more defined columnar morphology [24]. Another parameter that has an influence on the copper grain is the sputtering power. At higher sputtering powers, larger grains form as reported by Sun et al. [27]. For the applied preparation conditions, the copper columns obtained have a length of 2.52 µm ± 0.23, and the mean size diameter is 121.0 nm ± 27.2, as shown in the histogram in Figure 1D,E. A top-view analysis of the size of the copper columns was also performed (see Supplementary Materials Figure S1), showing that the sizes of the diameters analyzed in the top-view SEM images are correlated with the dimensions of the columns measured in the cross-section. As determined using the SEM analysis, the diameters of the columns synthetized via DC magnetron sputtering at a pressure of 4.0 × 10^−2^ mbar are in the nanometer scale. This nanocolumnar structure increases the active surface area of the deposit, making it suitable for sensing applications [26]. The EDX analysis carried out on the cross-section (see Figure 1C) revealed the presence of copper and oxygen, the latter having a content below 7 at.%. A maximum of 8% content of oxygen in the columnar copper layer has also been reported by Borisevich et al. [28].

**Figure 2:**
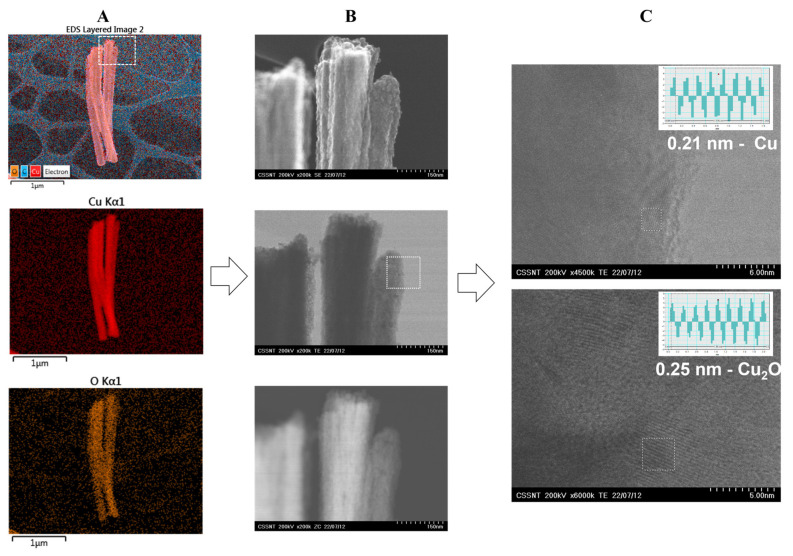
HR-STEM micrographs on CuNCs: (**A**) EDX elemental analysis; (**B**) SEM, TEM, and ZC investigations; and (**C**) interplanar spacing of 0.21 and 0.25 nm assigned to Cu and Cu_2_O, respectively.

To further analyze the fabricated material, HR-STEM images were recorded, as shown in Figure 2. The columnar structures were crystalline, and the interplanar distances of 0.21 and 0.25 nm were attributed to the (111) planes of Cu and Cu_2_O, respectively [29]. It is worth mentioning that the content of oxygen was low, i.e., 6.33 at.%, indicating that the NCs presented a small degree of oxidation. The columns were prepared under an argon atmosphere with an oxygen content below 2 vpm. However, this low content of oxygen may induce the formation of Cu_2_O, or it could be formed during the early period of exposure to oxygen after the deposition experiment is finished [30]. The elemental analysis indicated a uniform distribution of oxygen among the NCs. The HR-STEM analysis showed that the columnar structures were not made out of single crystal grains.

As mentioned in the Introduction Section, DC magnetron sputtering represents a simple, economical way to prepare copper with a nanocolumnar structure [18]. This method is highly reproductible, and in a few hours, it allows for the preparation of a large amount of electrodes covered with this nanomaterial. Copper-based nanostructures have found a variety of applications [31,32], and in this work, we focused on their use as glucose sensors. As presented above, the copper columnar structures present a small degree of oxidation as Cu_2_O, which could be beneficial for glucose sensing, since several authors have reported non-enzymatic glucose sensors based on copper oxide species [2,12,33]. 

### 3.2. Electro-Oxidation of Glucose on Cu Nanocolumns 

**Figure 3:**
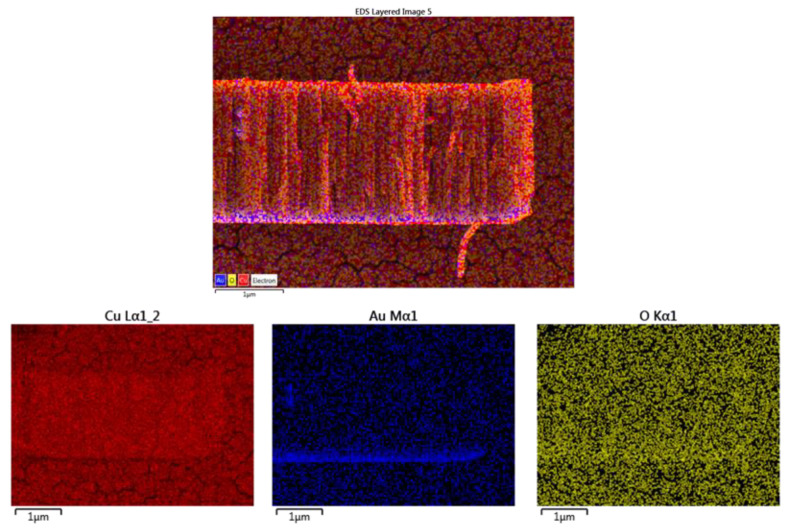
Cross-section EDX analysis of Au-CuNC.

For the electrochemical measurements, in order to improve the adherence of the Cu-NCs to the glassy carbon substrate (GC) in alkaline environments (see Supplementary Materials Figure S2), a nanometric-sized (~30 nm) layer of gold was deposited using *e*-beam evaporation prior to the fabrication of the copper via DC sputtering (Au-CuNC). The layer of gold is evidenced in the EDX mapping presented in Figure 3.

**Figure 4:**
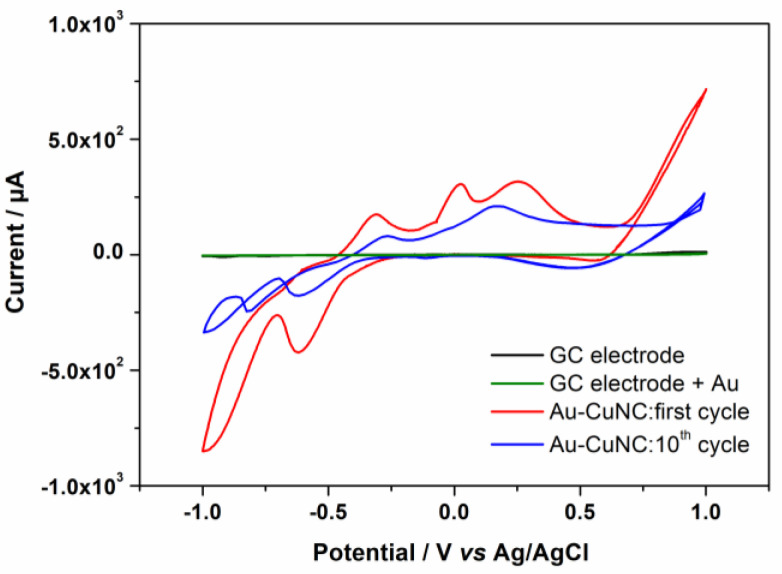
Cyclic voltammograms of GC electrode, GC electrode covered with a layer of gold, and the Au-CuNC electrode at 5 mV/s representing the 1st and 10th cycles in in 50 mM NaOH.

Prior to the sensing experiments, the behavior of the Au-CuNC was investigated in alkaline environments using cyclic voltammetry. Alkaline environments were selected because, in these types of media, the response of glucose is enhanced [6]. The experiments were performed in 50 mM NaOH solutions, and the results of the 1st and 10th cyclic voltammograms recorded at 5 mV/s are shown in Figure 4. As can be seen, within the investigated potential range, no electrochemical process occurred for the bare electrode or the one covered with only a layer of gold, which was expected for these inert surfaces. For the Au-CuNC, when scanning the potential in the anodic direction, three successive peaks appeared, with the first being T, assigned to the oxidation of metallic copper, Cu(0), to Cu(I) (at −0.33 V), and the peak at −0.004 V corresponding to the transitions of Cu(0)/Cu(II) and Cu(I)/Cu(II) [6,14]. The broad oxidation peak at around 0.25 V was considered to be related to the formation of Cu(III)-soluble species, such as CuOOH and Cu(OH)_4_^−^, which are further involved in the electro-oxidation of glucose [15]. In the cathodic scan, the peaks at 0.60 V, −0.59, and −0.91 V correspond to the reduction of Cu(III) to Cu(II), Cu(II) to Cu(I), and Cu(I) to Cu(0), respectively [14]. After multiple cyclic voltammograms, all the peaks exhibited a gradual decrease in the current.

**Figure 5:**
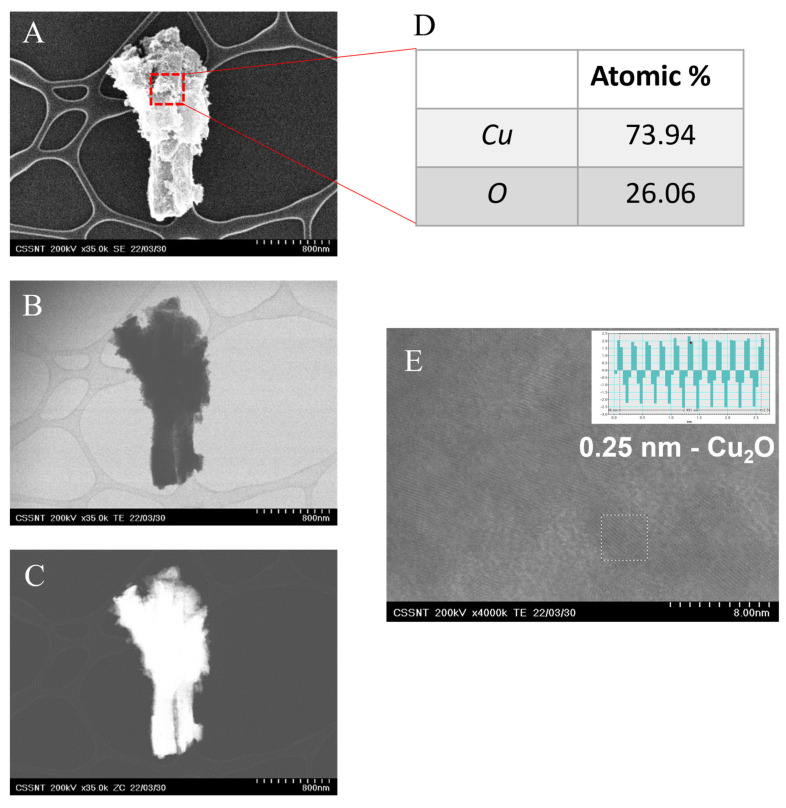
HR-STEM micrographs in (**A**) SEM, (**B**) TEM, and (**C**) ZC mode; (**D**) EDX analysis and (**E**) interplanar space determination.

The HR-STEM micrographs (see Figure 5) of the electrode exposed to ten cyclic voltammograms in 50 mM NaOH show that the CuNCs are covered by corrosion products [34]. The content of oxygen in the EDX analysis increases from 7 to 26 at.%. The film observed is crystalline, with an interplanar distance of 0.25 nm, assigned to the (111) orientation of Cu_2_O.

**Figure 6:**
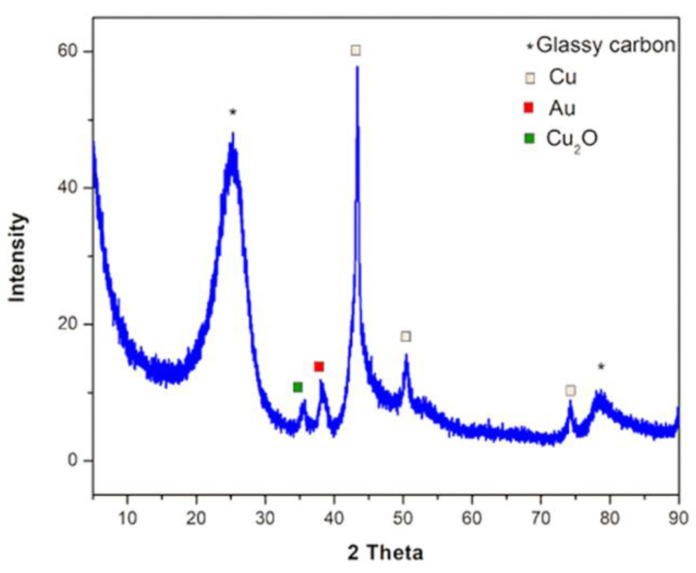
X-ray diffraction on Au-CuNC after 10 cyclic voltammograms at 5 mV/s in 50 mM NaOH.

In addition, the XRD pattern (Figure 6) also confirms the formation of Cu_2_O due to the presence of the peak at 35 deg., attributed to the (111) plane with an interplanar distance, 0.25 nm, identical to the one determined using HR-STEM. Furthermore, apart from Cu_2_O, metallic copper is identified. The peak ascribed to metallic copper, 43.3 deg., still exhibits the highest intensity in the X-ray diffraction spectrum, suggesting partial oxidation of the copper nanocolumns. After cyclic voltammetry is performed, the surface of the electrode is modified in an irreversible way with spongy-like corrosion products. Therefore, it can no longer be used in further investigations. 

The Au-CuNC electrodes were characterized using cyclic voltammetry in a solution of Fe(CN)_6_^4−/3−^ (see Supplementary Materials Figure S3) in order to determine the electroactive area (A_ea_), the roughness factor (*ρ*), the well-known kinetic parameter, the heterogeneous electron transfer constant (k_0_), and the standard heterogeneous rate constant (k_s_), which are reported in Supplementary Materials Table S1 and Figure S3. For comparison, an analogous analysis was performed on a Cu strip.

**Figure 7:**
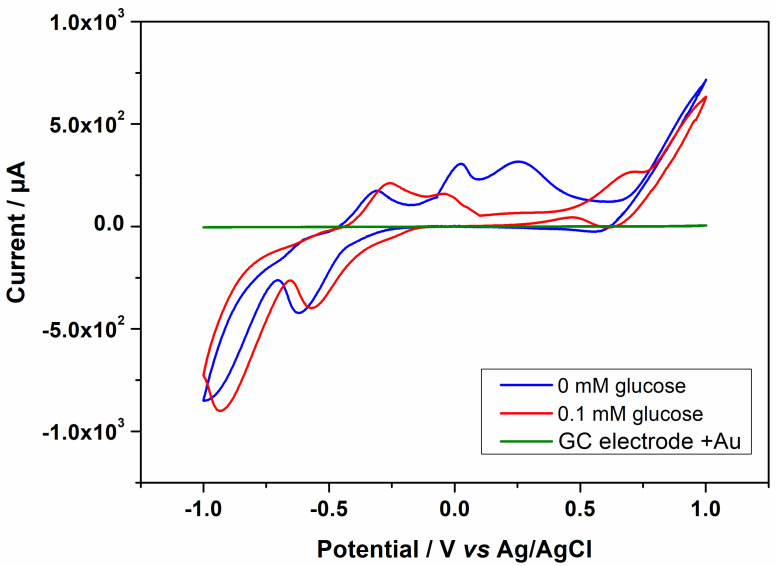
Cyclic voltammogram of the Au-CuNC without glucose and the GC electrode covered with Au and Au-CuNC in the presence of 0.1 mM glucose in 50 mM NaOH solution at 5 mV/s scan rate.

In the presence of 0.1 mM of glucose, the cyclic voltammogram shows several differences from that displayed in Figure 4. For instance, the broad peak associated with the formation of soluble Cu(III) species disappears, while the peak assigned to the electro-oxidation of glucose to gluconolactone appears at ~0.68 V [15,16,35]. Even though the mechanism behind the glucose oxidation process has not been clearly established, it is commonly believed that it occurs in the process of forming Cu(III) from Cu(II), where the Cu(III) species act as an electron delivery carrier [9,14]. The positions of the other peaks remain nearly unchanged. Moreover, as observed in Figure 7, the presence of the gold layer on the glassy carbon electrode does not influence the glucose oxidation process. The CV obtained for the copper strip in the presence of 0.1 mM glucose is presented in Supplementary Materials Figure S4, where it can be observed that the signal for glucose oxidation is lower for the copper strip than for the Au-CuNC electrode. 

**Figure 8:**
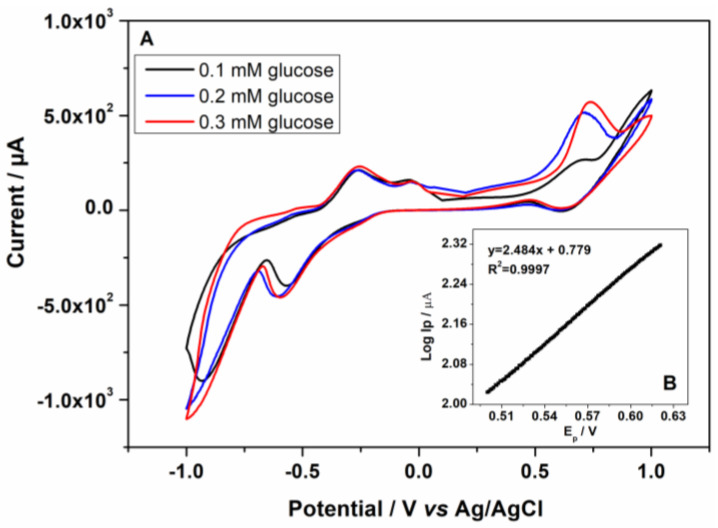
(**A**) Cyclic voltammograms of the Au-CuNC electrodes in the presence of 0.1, 0.2, and 0.3 mM glucose in 50 mM NaOH solution at 5 mV/s scan rate. (**B**) Tafel plot derived from the rinsing part of the CV obtained for glucose oxidation in 0.1 mM glucose in 50 mM NaOH solution at 5 mV/s.

The response of the Au-CuNC electrode to the further increase in the concentration of glucose up to 0.3 mM led to an increase in the oxidation peak of glucose and its slight shift to more positive values from 0.68 V to 0.72 V, as can be seen in Figure 8A. The electron transfer coefficient of the electro-oxidation process (α) was calculated from the slope of log I_p_ vs. E_p_ in the presence of 0.1 mM of glucose at a scan rate of 5 mV/s in 50 mM NaOH, as shown in Figure 8B. The value of α was found to be 0.85 [36]. 

Furthermore, in slightly alkaline environments, i.e., 5 mM NaOH, the electrochemical processes associated with glucose oxidation were not observed using cyclic voltammetry (see Supplementary Materials Figure S5). Therefore, 50 mM NaOH solution was selected for glucose testing. However, as previously shown in 50 mM NaOH solution, after performing CV on the electrode, its surface was covered with corrosion products, containing mainly Cu_2_O. Cyclic voltammetry in the presence of 0.1 mM glucose was performed on the electrode after it was exposed to 10 CV cycles, as seen in Supplementary Materials Figure S6, and no signal for glucose oxidation was observed. Therefore, the electrodes can no longer be used for glucose detection after performing CVs.

### 3.3. Amperometric Response to Glucose

**Figure 9:**
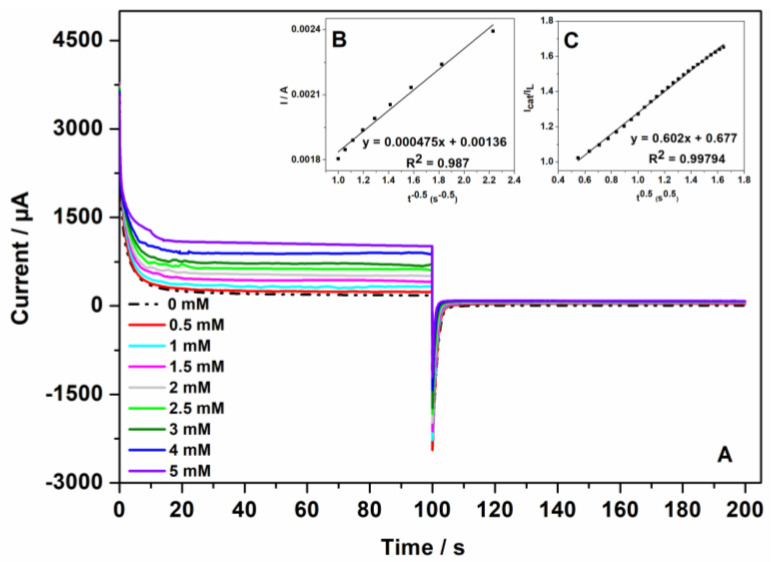
(**A**) Double-step chronoamperograms of Au-CuNC electrode in 50 mM NaOH for different glucose concentrations: 0, 0.5, 1.0, 1.5, 2.0, 2.5, 3.0, 4.0, and 5.0 mM (step potentials: +0.7 V and +0.25 V vs. Ag/AgCl); (**B**) dependency of I on t ^−1/2^; and (**C**) dependency of I_cat_/I_L_ on t^1/2^.

Double-step chronoamperograms were recorded on the Au-CuNC electrode in the absence and presence of different concentrations of glucose, from 0.5 to 5 mM, at two potential steps, namely, +0.7 V (value selected based on the previous cyclic voltammogram analysis), where the electro-oxidation process of glucose occurs, and at +0.25 V (see Figure 9A). When +0.25 V was applied, the value of the current was negligible. The diffusion coefficient of glucose was determined according to the Cottrell Equation (1):

I = nFACD^1/2^ π^−1/2^ t^−1/2^ (1)

where n is the number of transferred electrons, F is the faraday number, A is the area of the electrode, C is the concentration of the glucose, and D is the diffusion coefficient. From the slope of the resulting plots of I vs. t^−1/2^ (see Figure 9B) at different glucose concentrations, the average diffusion coefficient was calculated to be 4.25 × 10^−4^ cm^2^ s^−1^ [37]. 

Additionally, the catalytic rate constant was evaluated from the chronoamperometry experiments according to the following Equation (2):

(2) IcatIL=π12 (kcatCt)1/2

where I_cat_ are the currents in the presence of glucose, and I_L_ are the ones in the absence of glucose. C is the bulk concentration of glucose, and k_cat_ represents the catalytic rate constant. The mean value of k_cat_ was determined from the slope of I_cat_/I_L_ vs. t^1/2^ (see Figure 9C) at different glucose concentrations. k_cat_ was calculated to be 3.83 × 10^4^ cm^3^ mol^−1^ s^−1^ [38].

**Figure 10:**
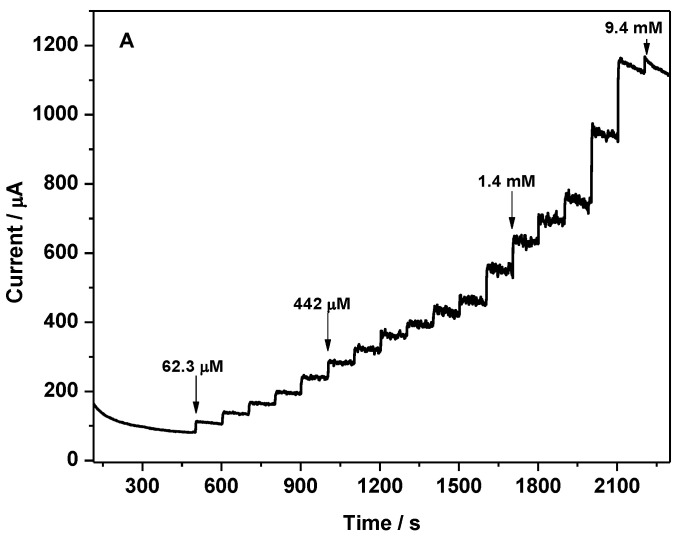

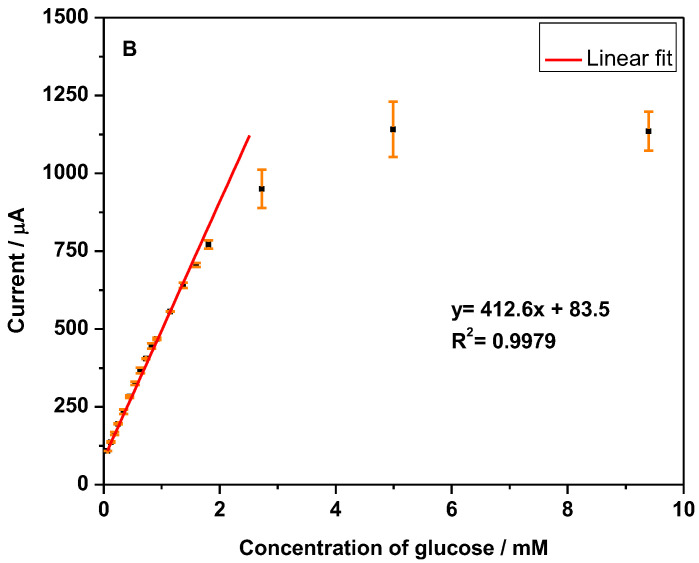
(**A**) Amperometric response of Au-CuNC in 50 mM NaOH to the successful addition of glucose from 62.3 uM to 9.4 mM at the applied potential of +0.7 V vs. Ag/AgCl; (**B**) the corresponding calibration curve.

The amperometric response of the Au-CuNC electrode for non-enzymatic glucose sensing was investigated using successive injections of glucose, from 62.3 µM to 9.4 mM, into 50 mM NaOH solution. The current–time dependences were recorded (see Figure 10A) when imposing the potential of +0.7 V, a value selected based on the previous cyclic voltammogram analysis. As the glucose concentration increased, the response current increased until the saturation value was reached at higher concentrations of glucose. In the calibration curve shown in Figure 10B, it can be seen that the sensor exhibits a linear range of up to 2 mM glucose, with R^2^ = 0.9979, a sensitivity of 21.02 µA/mM·cm^−2^, and a limit of detection (LOD) of 5.2 µM, values calculated based on the 3σ/slope equation, where σ represents the standard deviation of the blank. Actually, the LOD reported in this work is lower than that of other copper-based glucose sensors, such as Cu(II)/rGO/SPCE (65 µM) [17], Cu nanobelts (10 µM) [10], Cu-DA (20 µM) [39], Cu_2_O/PtE (26 µM) [40], Cu_2_O/Cu microstructures (37 µM) [33], CuNOx thin films (94.21 µM) [41], CuO nanowires (10 µM) [2], CuO nanorods/Nafion/GCE (23 µM) [12], and CuO nanoplatelets/Nafion/GCE (29 µM) [12]. Copper-based glucose sensors with an LOD lower than 5.2 µM have been reported in other works [9,15,35]; however, more often, their synthesis involves laborious processes and the use of hazardous products or elevated temperatures, not suitable for mass production, compared to DC sputtering, which is a clean and easy-to-scale-up method. Therefore, the prepared Au-CuNC electrode displayed a low LOD, and, in addition to its preparation advantages, it is promising for application in commercial glucose sensors. Finally, after performing amperometric investigations on Au-CuNC, no corrosion products were observed on the electrodes since, during this analysis, the potential was fixed at + 0.7 V and not varied as in CV.

### 3.4. Selectivity towards Glucose

**Figure 11:**
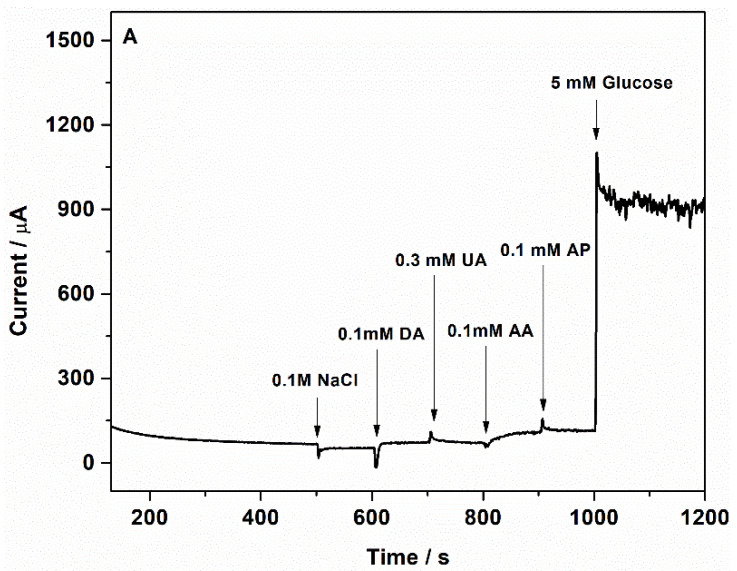

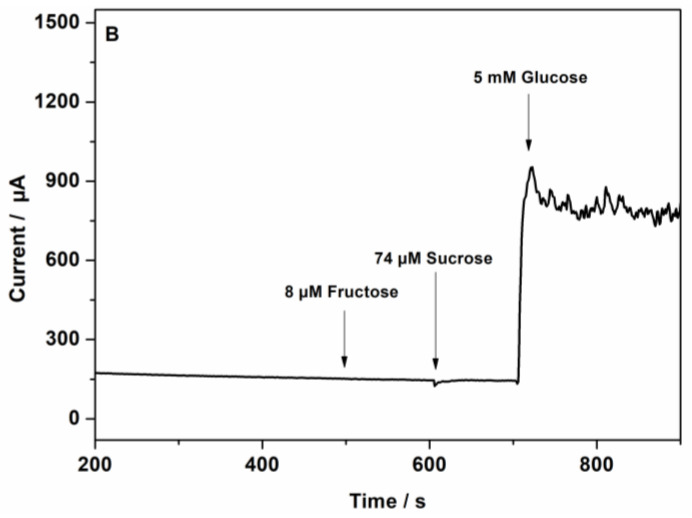
Amperometric responses of Au-CuNC to (**A**) 0.1 M sodium chloride, 0.1 mM dopamine, 0.3 mM uric acid, 0.1 Mm ascorbic acid, 0.1 mM acetaminophen, and 5 mM glucose in 50 mM NaOH and (**B**) 8 µM fructose, 74 µM sucrose, and 5 mM glucose in 50 mM NaOH.

In physiological samples, glucose coexists with other species, such as chloride ions, dopamine (DA), uric acid (UA), ascorbic acid (AA), and acetaminophen (AP). Therefore, the selectivity of the sensor towards glucose is important. Chloride ions, abundant in human blood, are considered one of the major poisoning species for non-enzymatic metallic-based glucose sensors [42]. The anti-interference properties of Au-CuNC sensors were examined using an orderly injection of normal physiological concentrations of NaCl (0.1 M), DA (0.1 mM), UA (0.3 mM), AA (0.1 mM), AP (0.1 mM), and 5 mM glucose to 50 mM NaOH solution at + 0.7 V. Neither the addition of NaCl nor the addition of other compounds presented a significant current response compared to glucose, as seen in Figure 11A. Furthermore, the presence of other sugars, such as fructose (8 µM) and sucrose (74 µM), at their physiological levels did not interfere in the glucose detection (Figure 11B). Consequently, Au-CuNC electrodes can be used in physiological glucose detection.

## 4. Conclusions

In this work, a clean and scalable technique, DC-MS, was presented for the preparation of copper nanocolumnar electrodes for non-enzymatic glucose sensing. The nanocolumnar features exhibited a mean size diameter of 121.0 nm ± 27.2 and a length of 2.52 µm ± 0.23. HR-STEM images show the formation of (111) Cu and Cu_2_O with interplanar distances of 0.21 and 0.25 nm, respectively. The behavior of the electrodes in 50 mM NaOH solution was investigated. After performing cyclic voltammetry (CV), it was observed that the surfaces of the electrodes were modified by the formation of corrosion products assigned to Cu_2_O, as revealed by the HR-STEM and XRD analyses. The electrocatalytic activity of the electrodes towards glucose oxidation was demonstrated by CV and chronoamperometry in alkaline environments. In CV, with an increase in the glucose concentration from 0.1 to 0.3 mM, the peak associated with glucose oxidation increased. The diffusion coefficient of glucose and the catalytic rate constant were calculated to be 4.25 × 10^−4^ cm^2^ s^−1^ and 3.83 × 10^4^ cm^3^ mol^−1^s^−1^, respectively, based on the chronoamperometric study. The amperometric responses of the electrodes were assessed by successively adding glucose at a fixed potential of +0.7 V vs. Ag/AgCl reference. A linear range of up to 2 mM and a limit of detection of 5.2 µM were achieved. The electrodes showed good selectivity towards glucose since an insignificant response was shown when physiological concentrations of dopamine, uric acid, ascorbic acid, and acetaminophen were added. In addition, the electrodes did not exhibit chloride poisoning. Furthermore, with their preparation advantages, the Au-CuNC electrodes displayed low LOD values, indicating that they are a promising option for commercial glucose sensors.

## Data Availability

Not applicable.

## Supplementary Materials

The following supporting information can be downloaded at: https://www.mdpi.com/article/10.3390/nano12234144/s1, Figure S1: Top-view diameter distribution; Figure S2: Images of electrodes after exposure to 50 mM alkaline environment: (a) Cu-NC on GC electrode; (b) Au-Cu-NC on GC electrode; Figure S3: (A) CV on Au-CuNC (red) and Cu strip electrodes (blue) at 50 mV/s in 5 mM [Fe(CN)_6_]^3−/4−^, 50 mM NaOH (inset Ipa vs. v^1/2^ for both electrodes); (B) plot of Ψ vs. 32.8 V^−1/2^ in 5 mM [Fe(CN)_6_]^3−/4−^, 50 mM NaOH. The factor 32.8 in the abscissa represents the quantity (π DnF/(RT)]^−1/2^. The inset presents the plots of Ψ values vs. ∆E_p_ for both electrodes, Figure S4: Cyclic voltammogram of Au-CuNC (blue) and Cu strip (red) in the presence of 0.1 mM glucose in 50 mM NaOH solution at 5 mV/s scan rate, Figure S5: Cyclic voltammetry of 0.1 mM of glucose on Au-CuNC in different alkaline environments, 5 mM and 50 Mm NaOH solutions, Figure S6: 10th cycle of CV of Au-CuNC electrode in 50 mM NaOH at 5 mV/s (red) and Au-CuNC electrode after 10th cycle in the presence of 0.1 mM glucose in 50 mM NaOH at 5 mV/s (blue). Table S1: Electrochemical parameters of electroactive area (A_eas_), roughness factor (ρ), heterogenous electron transfer constant (K_0_), and the standard heterogeneous rate constant (Ks) for Au-CuNC and Cu strip electrodes in 5 mM [Fe(CN)_6_]^3−/4−^ in 50 mM NaOH.
