# Supplementary material for: Synthesis of Copper Nanostructures for Non-Enzymatic Glucose Sensors via Direct-Current Magnetron Sputtering
Source: Nanomaterials (Basel). 2022 Nov 23;12(23):4144. doi: 10.3390/nano12234144 (PMC9739512; doi:10.3390/nano12234144)
Supplement: Supplementary file 1 [file nanomaterials-12-04144-s001.zip › nanomaterials-2005193-supplementary.pdf]

Supporting Information

Synthesis of Copper Nanostructures for Non-Enzymatic Glucose Sensors via Direct-Current Magnetron Sputtering

Sabrina State (Rosoiu) [1], Laura-Bianca Enache [1], Pavel Potorac [1], Mariana Prodana [2] and Marius Enachescu [1,3,*]

[1] Center for Surface Science and Nanotechnology, Politehnica University of Bucharest, Splaiul Independentei 313, 060042 Bucharest, Romania
[2] Department of General Chemistry, Faculty of Applied Chemistry and Materials Science, Politehnica University of Bucharest, Splaiul Independentei 313, 060042 Bucharest, Romania
[3] Academy of Romanian Scientists, Splaiul Independentei 54, 050094 Bucharest, Romania
* Correspondence: marius.enachescu@cssnt-upb.ro; Tel.: +40-21-4029100; Fax: +40-21-3181001

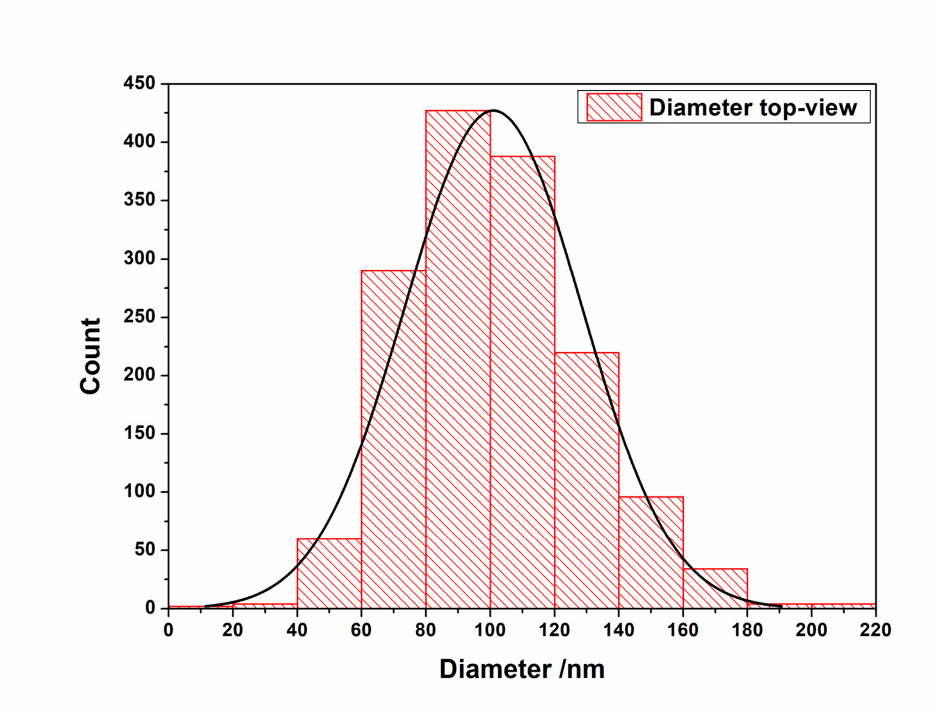

Figure S1. Top-view diameter distribution.

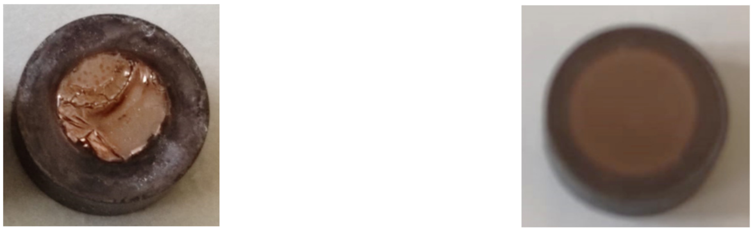

A B

Figure S2. Images of electrodes after exposing to 50 mM alkaline environment: A) CuNC on GC electrode; B) Au-CuNC on GC electrode.

Table S1. Electrochemical parameters of electroactive area (A_{eas}), roughness factor (ϱ), heterogenous electron transfer constant (k_o) and the standard heterogeneous rate constant (k_s) for Au_CuNC and Cu strip electrodes in 5 mM $[Fe(CN)_6]^{3-/4-}$ in 50 mM NaOH.

	A_{eas} / cm^2	ρ	k_o / $cm\ s^{-1}$	k_s / $cm\ s^{-1}$
**Au_CuNC**	0.26	1.32	$3.02 \cdot 10^{-3}$	$1.87 \cdot 10^{-3}$
**Cu strip**	0.20	1.02	$7.55 \cdot 10^{-4}$	$1.62 \cdot 10^{-3}$

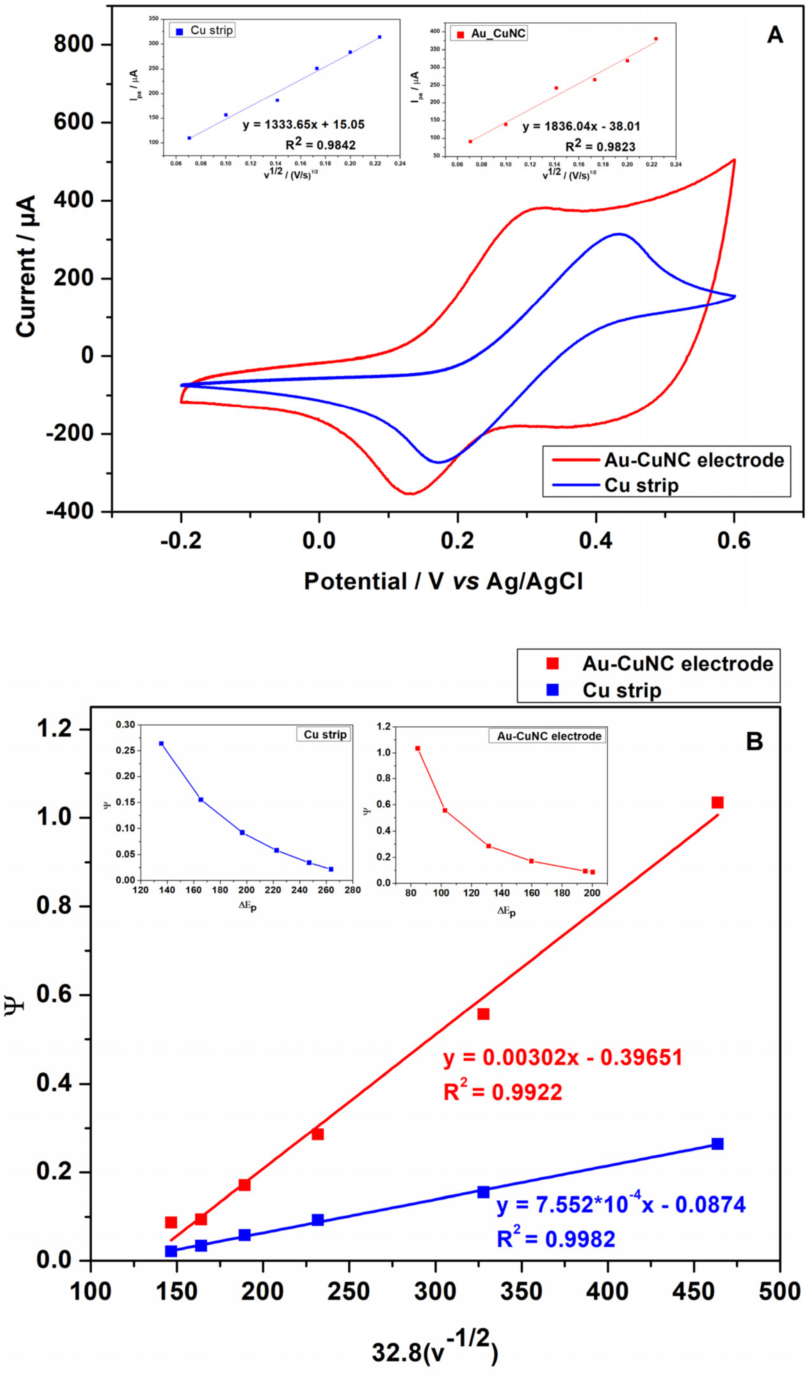

Figure S3. (A) CV on Au-CuNC (red) and Cu strip electrodes (blue) at 50 mV/s in 5 mM $[Fe(CN)_6]^{3-/4-}$, 50 mM NaOH (inset I_{pa} vs $v^{1/2}$ for both electrodes); (B) Plot of Ψ vs $32.8v^{-1/2}$ in 5 mM $[Fe(CN)_6]^{3-/4-}$, 50 mM NaOH .The factor 32.8 in the abscissa represents the quantity $(\pi\ DnF/(RT)]^{-1/2}$. The inset presents the plots of Ψ values vs ΔE_p for both electrodes.

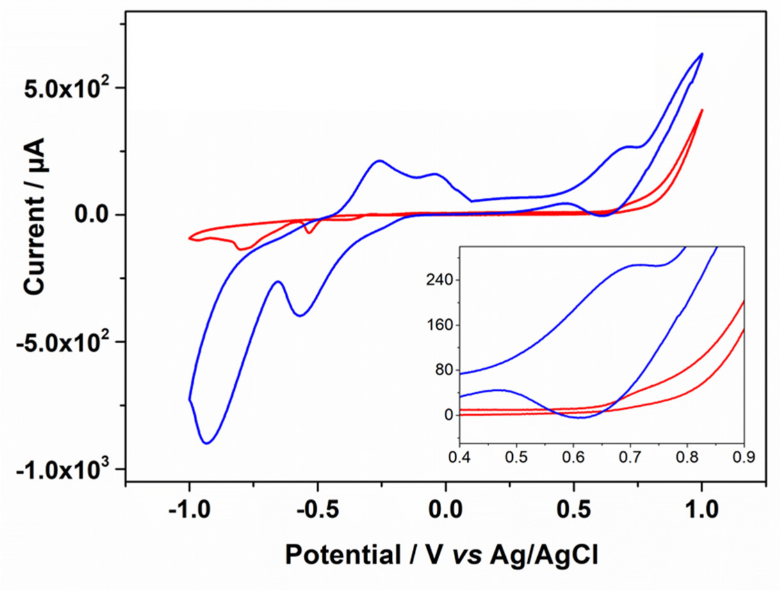

Figure S4. Cyclic voltammogram of Au-CuNC (blue) and Cu strip (red) in the presence of 0.1 mM glucose in 50 mM NaOH solution at 5 mV/s scan rate.

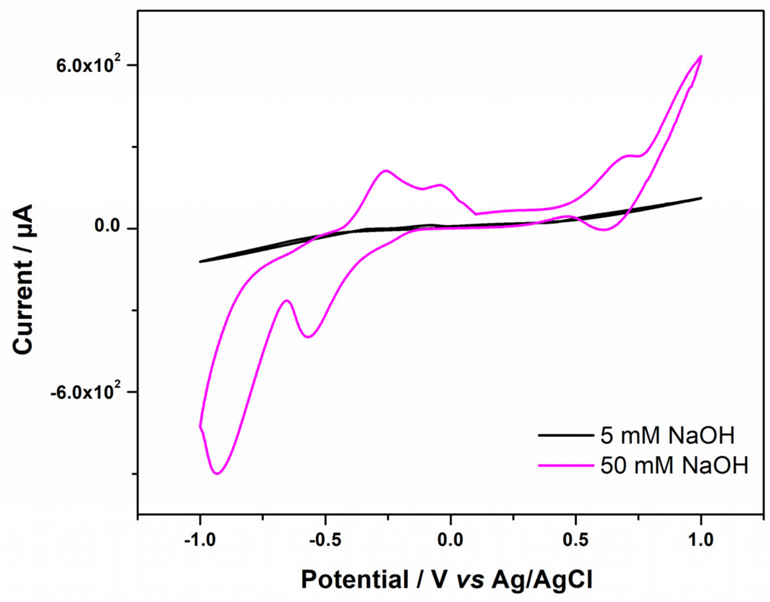

Figure S5. Cyclic voltammetry of 0.1 mM of glucose on Au-CuNC in different alkaline environments 5 mM and 50 Mm NaOH solutions.

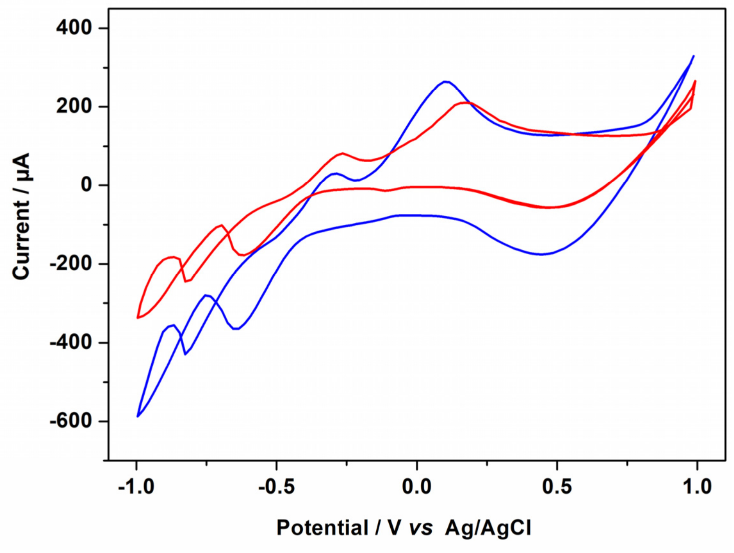

Figure S6. 10th cycle of CV of Au-CuNC electrode at in 50 mM NaOH at 5 mV/s (red) and Au-CuNC electrode after 10th cycle in the presence of 0.1 mM glucose in 50 mM NaOH at 5 mV/s (blue).
